# Study Protocol: Using Deep-Brain Stimulation, Multimodal Neuroimaging and Neuroethics to Understand and Treat Severe Enduring Anorexia Nervosa

**DOI:** 10.3389/fpsyt.2018.00024

**Published:** 2018-04-06

**Authors:** Rebecca J. Park, Jessica C. Scaife, Tipu Z. Aziz

**Affiliations:** ^1^Department of Psychiatry, Warneford Hospital, University of Oxford, Oxford Health NHS Foundation Trust, Oxford, United Kingdom; ^2^Nuffield Department of Clinical Neurosciences, University of Oxford, Oxford, United Kingdom

**Keywords:** anorexia nervosa, treatment, clinical trial, compulsivity, reward, deep-brain stimulation

## Abstract

**Background:**

Research suggests that altered eating and the pursuit of thinness in anorexia nervosa (AN) are, in part, a consequence of aberrant reward circuitry. The neural circuits involved in reward processing and compulsivity overlap significantly, and this has been suggested as a transdiagnostic factor underpinning obsessive compulsive disorder, addictions and eating disorders. The nucleus accumbens (NAcc) is central to both reward processing and compulsivity. In previous studies, deep-brain stimulation (DBS) to the NAcc has been shown to result in neural and symptomatic improvement in both obsessive compulsive disorder and addictions. Moreover, in rats, DBS to the NAcc medial shell increases food intake. We hypothesise that this treatment may be of benefit in severe and enduring anorexia nervosa (SE-AN), but first, feasibility and ethical standards need to be established. The aims of this study are as follows: (1) to provide feasibility and preliminary efficacy data on DBS to the NAcc as a treatment for SE-AN; (2) to assess any subsequent neural changes and (3) to develop a neuroethical gold standard to guide applications of this treatment.

**Method:**

This is a longitudinal study of six individuals with SE-AN of >7 years. It includes an integrated neuroethical sub-study. DBS will be applied to the NAcc and we will track the mechanisms underpinning AN using magnetoelectroencephalography, neuropsychological and behavioural measures. Serial measures will be taken on each intensively studied patient, pre- and post-DBS system insertion. This will allow elucidation of the processes involved in symptomatic change over a 15-month period, which includes a double-blind crossover phase of stimulator on/off.

**Discussion:**

Novel, empirical treatments for SE-AN are urgently required due to high morbidity and mortality costs. If feasible and effective, DBS to the NAcc could be game-changing in the management of this condition. A neuroethical gold standard is crucial to optimally underpin such treatment development.

**Clinical Trial Registration:**

The study is ongoing and registered with www.ClinicalTrials.gov, https://clinicaltrials.gov/ct2/show/NCT01924598, 22 July, 2013. It has full ethical and HRA approval (Project ID 128658).

## Introduction

Anorexia nervosa (AN) is one of the most challenging psychiatric disorders to treat, and its mortality rate is the highest amongst the psychiatric disorders (1, 2). It becomes severe and enduring anorexia nervosa (SE-AN) in at least a third of cases. There remains a grave paucity of evidence-based psychological therapies for AN (3–5), and there are few psychopharmacological treatments of benefit (6–8). The lack of effective treatments for SE-AN leads to huge morbidity costs to individuals and health-care services (9), so the development of novel, empirically-based and effective treatments is of major importance. This study investigates whether deep-brain stimulation (DBS) of the ventral anterior limb of the internal capsule (ALIC) within the nucleus accumbens (NAcc) is acceptable, feasible and helpful for adults with SE-AN.

DBS is a reversible, adjustable, non-destructive intervention using a surgically implanted medical device, which delivers carefully controlled electrical pulses to precisely targeted brain areas. While the exact mechanisms of action remain unclear, it has been suggested that DBS works by rebalancing resting-state networks (RSNs) in the brain. Neuroimaging studies have demonstrated that RSNs are abnormal in AN (10–12). In this study, we will use magnetoencephalography (MEG) to monitor post-operative changes in neural activity. Recordings will be made both in the resting state and also during a food reward task in which participants are asked how much they want high- vs low-calorie food stimuli. MEG scanning is a real-time measure of neural responses, which we have previously used to demonstrate attentional bias to food cues in a currently ill population (13).

DBS has been widely used, particularly for Parkinson’s disease, for over 30 years. Increasingly over the last decade, DBS has been applied to the treatment of psychiatric disorders including treatment-resistant depression (14–17), obsessive compulsive disorder (OCD) (18, 19) and addictions (20). The US Food and Drug Administration approved a humanitarian device exemption for DBS in the treatment of severe OCD in 2009. This resulted from a review of data from 26 patients with severe treatment-resistant OCD who underwent DBS to the ALIC/ventral striatum (VS). On average, after 12 months of therapy, there was a 40% reduction in patients’ OCD symptoms (21).

Two lines of evidence indicate that DBS might be effective in SE-AN. Firstly, OCD and AN are highly co-morbid, with both disorders showing a high degree of compulsivity (22–24). The second arises from case reports and series. A patient with severe OCD also found improvement in co-morbid anorexia following DBS (25). Further case reports and case series of DBS for SE-AN (26–28) suggest benefit, although none have included double-blind DBS on–off phases. The largest case series, of 6 patients with SE-AN (29) involved DBS applied to the subcallosal cingulate, a neural target previously used for treatment-resistant depression. Regarding safety, the authors initially reported one serious adverse event (SAE): a seizure 2 weeks post-operatively in the context of AN-related metabolic disturbance, with no permanent sequelae. A further patient had a panic attack intraoperatively, and another had an air embolism, but there were no long-lasting adverse effects of the surgery for either participant. A follow-up paper reported sustained symptomatic improvement and weight restoration in three out of six patients, possibly mediated by improvements in mood regulation (30). A recent single case study has also reported improvement in eating disorder psychopathology and mood in an individual with chronic AN, following DBS to the bed nucleus of the stria terminalis (28).

Individuals with AN experience an aberrant sense of reward from weight loss and self-starvation (9, 31, 32) with neural evidence that reward processing becomes abnormal (33, 34). Energy-dense food is anxiety-provoking and aversive, such that individuals become unable to eat to sustain life (35, 36). The compulsive pursuit of self-starvation in AN represents a major barrier to treatability, rendering contemporary treatments highly aversive and contributing to high dropout and relapse rates (31, 37). If these disturbed reward processes could be characterised and subsequently interrupted at a neural level, it would revolutionise the treatment of AN.

The NAcc, located deep in the VS, is central to reward processing and has been described as a “hedonic hotspot” [38]. In rats, DBS to the medial shell of the NAcc has been shown to increase food intake (39), and activity in this structure is key to experiencing food as rewarding (38). Neuroimaging and experimental studies in AN (31, 32, 37) confirm a dysregulation of reward circuitry: in particular, an abnormal striatal response to food and thinness cues (33, 34, 40). There is also evidence of an increased top-down control over reward circuitry in AN, emanating from the prefrontal cortex (41).

The relentless self-starvation and over-exercising seen in AN have parallels with OCD and addictions with evidence of aberrant reward processing in common with these other pathologies (22, 24, 42–45). There is evidence that compulsivity is a transdiagnostic process common across these disorders (23), mediated at a neural level by corticostriatal thalamic circuits, which incorporates the VA and NAcc (46). Excessive reliance on habit formation in learning has been suggested as the basis of persistent compulsive behaviour in OCD (47) and more recently in AN (9). It has been suggested that restrictive eating and weight loss in AN begin as “action-outcome/goal-directed learning” in which behaviour is associated with a rewarding outcome (44). However, through repetition, reward processing becomes aberrant such that the behaviour no longer relies upon reward reinforcement (stimulus–response learning), and this behaviour becomes highly resistant to change (48). This reduction in goal-directed learning in eating disorders has been demonstrated using a two-step habit formation task (49). This task will also be undertaken by participants in this study to record any changes in compulsive, habitual responding (50).

Given the high mortality and morbidity rates of SE-AN (51), additional risks to patients of surgery and entering this study are small, with a potential cost–benefit analysis that justifies ethical equipoise (52). Professor Tipu Aziz, who performs all the DBS operations, has an excellent personal safety record and has performed thousands of operation on patients with Parkinson’s disease. He has also operated on adults with chronic pain and children with movement disorders. The procedure itself has an overall risk of stroke of less than 1%, a haemorrhage-causing death of 0.01% and a risk of wound infection of 5%. To minimise the risk of surgery, the patients will be medically stable prior to surgery. They must have a normal ECG and electrolytes, be free of severe binging or purging or severe depression/suicidality, both of which pose added background risks. The technique (as performed in Oxford) is very well tolerated and can be done in one stage, supported by two to four nights as an inpatient in the neurosurgical ward. DBS has a one-off cost of approximately £40,000 to implant a rechargeable device with a 10-year lifespan. As the mechanisms, efficacy and optimal DBS target for intervention in AN are yet to be established, it should be regarded as an experimental treatment, with the risk that it will not be effective. However, given the extremely poor outcome in terms of morbidity and mortality for those with SE-AN, it is plausibly an intervention where the benefits may outweigh the risks and costs.

A significant body of research suggests that altered reward processing contributes to the maintenance of SE-AN and that this is influenced by higher cognitive control processes (9). We hypothesise that DBS to the NAcc may ameliorate aberrant reward processing and AN psychopathology.

In this clinical trial, our aims are to examine (1) the safety, acceptability and feasibility of DBS to the ALIC at the NAcc in SE-AN; (2) to map neural mechanisms and symptomatic change following DBS; (3) to explore ethical issues, capacity, consent and patients’ views pre- and post DBS and (4) to explore the longer-term effects of DBS in SE-AN.

## Methods

This is a study of DBS targeting the NAcc in up to six patients with SE-AN, using pre- and post-operative repeated measures. To date, five patients have been enrolled in the study.

**Figure 1:**
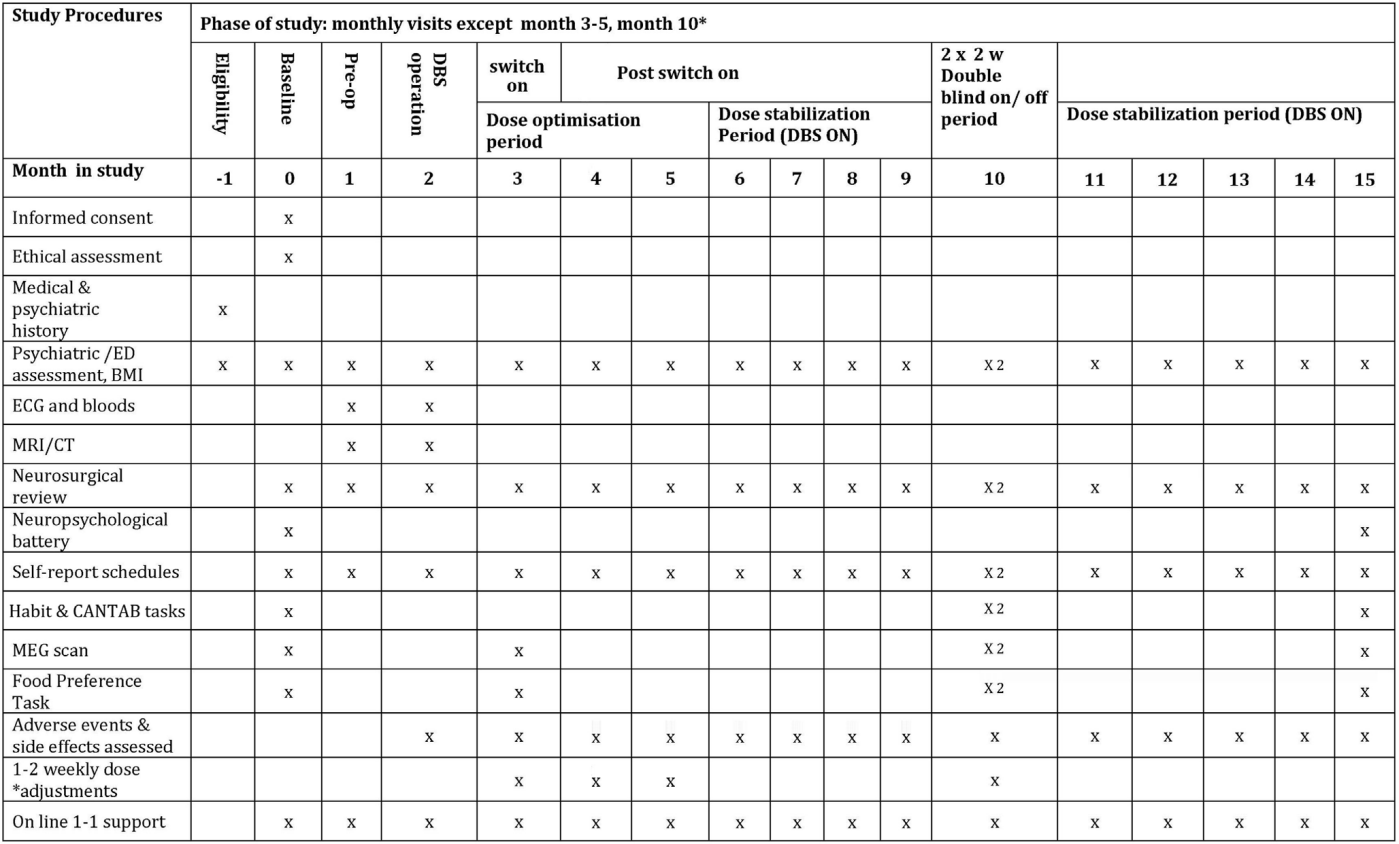
Schedule of study procedures.

The study period is 15 months—see Figure 1 for details of study interventions. There will be pre-operative assessments in month 1, the DBS operation in month 2, followed by the DBS switch-on at the end of month 3. DBS dose will be optimised during months 3–6. During month 10 of the study (6 months after switch-on), there will be two double-blinded 2-week periods of DBS on/off as described by Denys et al. (53). At 15 months (12 months after switch-on), there will be final follow-up tests. This study is a quantitative and qualitative investigation of eating disorder and co-morbid pathology, using MEG to image neural processes, and computerised tasks to assess habit formation. There is also a parallel quantitative and qualitative sub-study of ethical issues involved.

### Patient Population and Recruitment

#### Inclusion Criteria

- Primary diagnosis: AN according to the DSM-V criteria, based on a psychiatric interview
- Illness duration of severe AN of >7 years
- Disabling severity with substantial functional impairment
- Treatment refractoriness, defined as lack of response to two or more typical modes of treatment, such as inpatient weight restoration, psychotherapy and psychopharmacology
- Severely underweight: Body Mass Index (BMI) > 13 < 16
- 21–65 years old
- Written informed consent
- Able to fully understand the consequences of the procedure
- English speaking and able to answer the study questions fluently
- Has the mental capacity to provide informed consent to research participation

#### Exclusion Criteria

- Unstable physical condition (severe electrolyte disturbances, cardiac failure and other physical conditions due to low weight in which surgery/anaesthesia is contraindicated)
- Treatable underlying cause of anorexia/underweight
- Parkinson’s disease, dementia, epilepsy
- History of schizophrenia/psychosis, bipolar disorder
- Alcohol or substance abuse (including benzodiazepines) during the last 6 months
- Current severe major depressive or Tic disorder
- Antisocial or severe Borderline Personality Disorder
- Standard MRI scan exclusion criteria (pregnancy, pacemaker and metals contraindicated for MRI, except for the DBS implantation and stimulator itself)
- Currently an involuntary patient

### Patient Selection Process

The study patients will have SE-AN. Individuals fulfilling inclusion and exclusion criteria under the clinical care of specialist eating disorder services will be ranked in the order of suitability according to the below specific criteria:

- Degree of severity and intractability of AN
- Has never had periods of full remission
- Has tried existing treatment options without success
- Intensity of desire to recover
- Restrictive AN—without bingeing/purging
- Absence of currently severe co-morbid depression or self-harm
- Ideally on no, or minimal, psychotropic medication
- No recent history of compulsory treatment (within the last 12 months)
- Good intellectual function and education—well able to comprehend the facts of the intervention
- Poor social and occupational function, high levels of distress—not functioning well, has poor quality of life

A ranked list of potential participants will be made in consultation with direct clinical care consultants prior to Rebecca Park approaching patient(s) one by one in rank order. If the first is not suitable or not interested, the next ranked will be approached.

## Trial Sites

The study involves collaboration between the Department of Psychiatry and the Department of Surgery at the University of Oxford. Research activities will take place at the Department of Psychiatry, University of Oxford, Warneford Hospital and the Department of Neurosurgery, John Radcliffe Hospital, Oxford.

### Phases of the Study

#### Phase I: Screening and Enrolment

Suitable patients will be identified in close liaison with specialist eating disorders clinical teams who can consult Rebecca Park initially as to their suitability. If felt to be potentially suitable, the patient will first be informed of the opportunity to take part in the study by their clinical care team. If they show interest, they will be provided with a patient information sheet (PIS) and will meet with Rebecca Park initially for a preliminary assessment. If deemed suitable, the patient will then meet Rebecca Park and Tipu Aziz jointly for further information, either alone or with their family, with ample opportunity for questions. They will be given at least a week to decide whether to participate, before being consented by Rebecca Park. At all times, participants will be assured that their participation is entirely voluntary and that they may withdraw from the study at any point with no implications for their clinical care.

All patients will need to have normal electrolytes, and ECG and anaesthetic review prior to being considered fit for the operation. Patients need to have a BMI of over 13 for surgery.

#### Phase II: Pre-operative Baseline Evaluation

##### Assessment with Research Team (Repeated at All Subsequent Monthly Follow-ups)

Semi-structured interviews assessing psychiatric symptoms (including eating disorder symptoms) will be performed, along with the completion of self-report questionnaires which index eating disorder psychopathology, mood and participants’ quality of life. This is a battery of validated standardised questionnaires, of gold-standard use in studies of eating disorders and OCD, which will be used to assess mood, anxiety, obsessionality and eating disorder psychopathology:

- Eating Disorder Examination (EDE) (54)
- Global Assessment of Functioning DSM-IV (55)
- Structured Clinical Interview for DSM-5 (56)
- Hamilton Anxiety Rating Scale (57)
- Hamilton Depression Response Scale (58)
- Yale–Brown-Obsessive Compulsive Scale and checklist (59)
- Yale–Brown–Cornell Eating Disorder Scale (60)
- EDE Questionnaire (61)
- Self-Starvation Scale (62)
- Clinical Impairment Assessment (63)
- WHO Quality of Life Scale (64)
- Beck Depression Inventory (65)
- State Trait Anxiety Inventory (66)
- Snaith–Hamilton Pleasure Scale (67)
- Ruminative Response Scale for Eating Disorders (68)
- BMI is also calculated during the baseline assessment.

##### Neuropsychological Assessment

A neuropsychological assessment battery will be carried out by a clinical neuropsychologist. This comprises the following:

- WAIS-IV subtests: Vocabulary, Matrix Reasoning, Digit Span, Coding, Similarities (69)
- BIRT Memory and Information Processing Battery: List-Learning Task (70)
- Verbal Fluency (Baseline Measure of Phonemic and Semantic Fluency) (71)
- Rey Complex Figure task—assessment of the ability to copy, immediate recall, delayed recall (72)
- Iowa Gambling Task (73)
- Trail-Making Task Parts A & B (74)
- D-KEFS Colour Word Interference (75)
- Hospital Anxiety and Depression Scale (76).

##### Pre-operative Capacity Assessment and Ethical Sub-Study

Once each suitable patient is identified and consented, then Jacinta Tan, an independent psychiatrist and ethicist, will interview the patient and perform a complete MacCAT-CR assessment (77), along with an in-depth interview to explore the patient’s experience of treatment, rationale for participation and motivations for taking part in research. This follows the Oxford Neuroethics Research Paradigm which ensures a high level of capacity, voluntariness and informed consent. It is described in more detail in our recently published paper (52) and also on the registered trial online: https://clinicaltrials.gov/show/NCT01924598. We integrated this ethical sub-study from the inception of this protocol as recommended by recent consensus guidelines (78). It is guided by the foundational principles of the Nuffield Council of Bioethics report on “Intervening in the Brain” (79).

The assessment will take 2.5–3 h and can be done in sections if the patient feels tired. It will be tape-recorded and transcribed. Jacinta Tan and an independent observer will separately score the MacCAT-CR. Both will also provide global assessments of capacity to consent to research. Jacinta Tan will provide a detailed report of the interview and her assessment of ethical acceptability of participation for that individual. This will ensure that the needs of the research are not prioritised over those of the patient. If the patient is not suitable, the process will be repeated with the next interested and potentially suitable individual on the list.

##### Neuroimaging and Computer Tasks

Pre-operatively, patients will undergo the following:

- A whole-brain MRI scan including T1, T2 and Inversion Recovery sequences at the John Radcliffe Hospital, Oxford, with a total duration of 20 min.
- A whole-brain MEG scan: a resting state and a food-“wanting” task (13), which will be performed at OHBA, the Warneford Hospital, Oxford, with a duration of 20 min.
- Computerised behavioural tasks of reward: The Leeds Oxford Food Preference Questionnaire (LOFPQ) (35), 2-step habit formation task (50) and tasks from the Cambridge Neuropsychological Test Automated Battery (CANTAB) (80).

##### True Colours

Participants will complete weekly self-report symptom questionnaires at home (estimated to be 5 min) using the “True Colours” online self-monitoring system pioneered at the University of Oxford, Department of Psychiatry, adapted for Eating Disorders by Rebecca Park by including the OxBREaD brief-rating scale: http://oxtext.psych.ox.ac.uk/true-colours. This self-report system will continue from the study entry to the study end.

#### Phase III Operative Phase: Month 2

The intervention consists of bilateral DBS targeted at the NAcc with stimulation at the ventral ALIC. The stimulation can be programmed and adjusted non-invasively by a trained clinician to minimise side effects and maximise symptom control.

The DBS system consists of three components: two electrode leads that are inserted into the brain, the implanted pulse generator (IPG) that contains a battery and a circuitry to produce the stimulus current, and the extension leads that run subcutaneously to connect these together. All three components are surgically implanted inside the body, with the IPG typically placed subcutaneously in the pectoral or the abdominal region. Patients will be implanted with the Medtronic RC pacemaker and the 3387 electrode. After implantation, the IPG can be calibrated by a trained clinician to optimise symptom suppression and control side effects. A rechargeable pacemaker lasts for 10 years and is much smaller than a non-rechargeable one, so is ideal for those who are underweight.

The whole procedure will be completed in 1 day and in total will require up to 4 days and 3 nights of inpatient stay at the John Radcliffe Hospital. On the day of surgery, the patient will be anaesthetised and the base ring fixed to the skull and a CT scan performed with a localiser fixed to the ring. The patient will then be transferred to theatre and the scalp cleansed. By fusing the structural MRI scan to the stereotactic CT scan, the trajectory to implant electrodes into the NAcc bilaterally will be calculated. Then, through bilateral scalp incisions and a twist-drill skull perforation (2.5 mm in diameter), deep-brain electrodes will be passed to target bilaterally and fixed to the skull with titanium mini plates. The scalp will then be closed, and a repeat stereotactic CT scan will be obtained to confirm electrode placement.

Having done so, back in the theatre, the electrodes will be connected to extension cables that will then be passed subcutaneously down one side of the head, behind the ear to a subcutaneous pouch below the collar bone and connected to a rechargeable pacemaker and all the wounds closed. Video recordings are used as part of the assessment process during routine implantation of DBS wires, whether taking part in a study or not, and are therefore considered part of standard operating procedure.

The day after surgery, the pacemaker will be turned on to ascertain any immediate effects of stimulation on symptoms and then switched off again. The patient will then be discharged 2 days after implantation. The stitches will be removed and the wound checked after 2 weeks. They will be assessed a month later when all wounds are healed and any acute effects of the DBS electrode insertion operation such as headaches, scalp soreness or wound tenderness have worn off. If so, DBS will be switched on: patients will be turned on to a setting of deepest contact −ve, third contact +ve, amplitude 2.5 V, pulse width 60 ms and frequency 130 Hz. Patients will have monthly assessments, and by the third visit, they will be on a final amplitude of 4 V.

#### Phase IV: Post-operative Follow-up Phase

##### Post-operative Assessments, Aiming to Track the Neural and Symptomatic Change As a Result of DBS, Will Involve

*Weekly* Self-report-rating scales of eating disorder psychopathology, anxiety and depression involving the True Colours text-messaging system started in phase I: http://oxtext.psych.ox.ac.uk/true-colours (5 min) (see Phase II: True Colours) (see Figure 1 for more details). Weekly side effects questionnaires, the SAFTEE-SI (81) and DBS side effects questionnaire (82).

Monthly

- Interview with Rebecca Park and Jessica Scaife. Repeat of baseline measures collected pre-operatively [see Phase II: Assessment with Research Team (Repeated at All Subsequent Monthly Follow-ups)].
- Joint neurosurgery–psychiatry reviews with Rebecca Park and Tipu Aziz, senior nurse practitioner and Jessica Scaife. During this review, neurosurgical parameters (such as the degree of stimulation) and psychiatric parameters (such as experience of eating disorder and co-morbid psychiatric symptoms) will be jointly assessed. Any necessary adjustments to stimulator intensity will then be programmed.

Post-operative Neuroimaging

- Resting state and food “wanting” task under MEG (13), at OHBA, at month 3 (pre-DBS switch-on), two scans in month 10 in DBS on and off conditions and at the end of study in month 15. Post-operative MRI is not possible with the stimulator implanted for safety reasons.
- Following each MEG scan, participants will carry out the LOFPQ food reward task. Tasks from the CANTAB (80) and a task measuring habit formation (50) will be carried out twice during the DBS on-off phase and at the end of the study (see Figure 1).

Post-operative neuropsychological assessment at 15 months (12 months after DBS switch-on) will be a repeat of those tests detailed in Section “Phase II: Neuropsychological Assessment.”

#### Phase V: Ethics Sub-Study

The protocol for the ethics sub-study was recently published (52), giving best-practice guidelines for such research worldwide. A separate, optional post-operative ethics sub-study will also be offered. Participants will be informed about this option in the main study PIS. In the main study consent form, they will be asked if they agree to being contacted about the post-operative ethics sub-study.

It will involve one 2.5-hour session with an independent psychiatrist, Jacinta Tan, near the end of the post-operative follow-up period (after 6 months). The sub-study will have a separate PIS and separate informed consent form. This consent form will be requested for the participant’s permission to draw upon the assessment recordings and measures obtained during the main DBS study for use in the subsequent ethical research and analysis.

If the patients wish to be contacted regarding this ethics sub-study, they will be sent the information sheet during the follow-up period. If they consent to participation, the single post-operative interview will occur at the end of the post-operative follow-up period, in order to maintain Jacinta Tan’s roles as an advocate and a clinical researcher distinct.

The post-operative ethics interview will explore the ethical issues with the participant that surround the research. Jacinta Tan will elicit her experience of participation, her reflections on this, and also views in hindsight of the ethical issues surrounding the research from the participant.

At the end of the 15-month protocol period, the patient has the option to remove the DBS device which can be done in a simple operation with minimal risks. There will be the option for an annual research follow-up for up to 4 years. At the end of protocol period, or at any point subsequently, the patient has the option to remove the DBS device which can be done in a simple operation with minimal risks. If the participant decides to keep the DBS stimulator *in situ*, they will have routine neurosurgical DBS follow-up every 12 months for clinical care. The annual research follow-up if consented to would take place at the same time and include the following:

- The same questionnaires as completed monthly during the main study.
- The same computerised tasks as completed pre- and post operation.
- A FaceTime/Skype or phone interview covering psychiatric symptoms and individual experience.
- The same neuropsychological battery they completed pre-operatively.

## Safety

An SAE is any untoward medical occurrence that results in death, is life-threatening, requires inpatient hospitalisation or prolongation of existing hospitalisation, or results in persistent or significant disability/incapacity.

### Reporting Procedures for SAEs

“An SAE occurring to a participant should be reported to the REC that gave a favorable opinion of the study, where in the opinion of the Chief Investigator (CI) the event was: ‘related’ – that is, it resulted from administration of any of the research procedures; and ‘unexpected’ – that is, the type of event is not listed in the protocol as an expected occurrence. Reports of related and unexpected SAEs should be submitted within 15 days of the CI becoming aware of the event, using the NRES report of serious adverse event form (see IRAS/NRES website). All reports will be cc’d to the sponsor Clinical Trials & Research Governance, University of Oxford (CTRG)”.^1^

### Data Analysis

Number of participants: this is an exploratory study of up to six patients, and therefore a precise power calculation is not possible. External review of the protocol for an initial single patient pilot (by the international expert in the neuroscience of AN, Professor Kaye, University of San Diego) suggested the scientific importance of extending the study to include more patients.

Treatment effects will be established using within-subject analyses comparing baseline characteristics with patient assessment at each monthly follow-up session and by comparing change in symptoms during periods when the stimulator is “on” and periods when the stimulator is “off.” We plan to use multiple regression, analysis of variance and *t*-tests using SPSS software to analyse the data. Results can be compared to our published findings from a linked multimodal neuroimaging case–control study of reward processing in current AN, recovered AN and controls (12, 13, 36).

In addition, MEG data will be analysed using in-house software and open software packages. Preprocessing of data will rely on propriety software supplied by the scanner manufacturer. We will employ both signal and source space methods, building on our previous approach to analysing and interpreting neural data obtained in patients with AN. Non-parametric statistics and general linear modelling will be used to establish significance.

### Data Monitoring

Data monitoring for the project will be conducted by the CI and co-investigators. The study may also be audited by the CTRG. There will be no external monitoring of the study. The project will be under ongoing review for safety and efficacy by the CI and Dr. Park. The study may also be audited by the CTRG, with extensive protocols from the University of Oxford. Any SAE directly relating to the DBS electrode insertion and which cannot be immediately resolved will result in electively stopping the trial prematurely.

## Participant Confidentiality

The study team will ensure that the participants’ anonymity is maintained. A basic dataset of name, age, predominant symptoms and medications will be recorded. All electronic data will be anonymised with the date of recording and a unique project number as the only identifiers. It will be stored on secured university servers and is covered by the Oxford University Data protection register (reference No. Z575783X). All non-anonymised paper data (e.g., consent forms) will be stored in a locked cupboard within a secure building. All paper records will be kept in the research department at all times.

### Capacity and Informed Consent

Ethical issues are of major importance in the treatment of AN and will be the focus of an ethical sub-study (detailed in section Phase V: Ethics Sub-Study) (52). Only patients with full capacity, fully consenting and voluntarily entering into the study will be eligible.

### Burdens

The study does involve a number of return visits for outpatient assessment and follow-up, and includes up to 4 days spent in hospital for the DBS surgery in order for these assessments to be carried out. Therefore, we will aim to recruit patients who are physically mobile and are prepared and able to return for multiple assessments as outlined. However, involvement is entirely voluntary, and the patient can withdraw at any time.

## Discussion

We have presented an innovative clinical trial protocol, applying DBS research to individuals with SE-AN. We hope this will guide future researcher, ethics committees and the development of technologies designed for psychiatric populations. We recommend this protocol as a starting point to guide future trials. We further suggest that the neuroethical framework we have now published (52) should also be incorporated in such experimental treatment trials in SE-AN, due to unique concerns regarding physical wellness and capacity. The protocol we describe incorporates this ethical gold standard (52), which acts in synergy with the main trials protocol described here. It serves as means to investigate, empirically, the ethics of such an experimental treatment. Incorporating neuroethical standards centrally within novel neurotechnology research aims to ensure that rigorous and continuous ethical input is available. This is of great importance given the sensitive and challenging nature of this research, and it is essential that the welfare of vulnerable participants is maximally protected. Our protocol exemplifies the Nuffield Council of Bioethics’ recommendations regarding the ethics of novel neurotechnology (2013) (79). We believe that the clinical trial described here can contribute to treatment development for AN and the ethics of such research in addition to extending the boundaries of DBS science.

## Availability of Data and Materials

The authors can confirm that all relevant data are included in the article. Data generated by the protocol will be included in subsequent publications and/or supplementary information files once the study is completed. Dissemination will be by presentation at conferences and in peer-reviewed publications.

## Insurance

In the event of any participant suffering harm as a result of their involvement in the research, the University has a specialist insurance policy in place which would operate (Newline Underwriting Management Ltd., at Lloyd’s of London, policy numbered: WD1200463).

## Trial Status

The trial status is ongoing, and recruitment commenced on 8 October, 2013. Active recruitment had to be paused after the first patient due to MEG scanner refurbishment (4/15–4/16): protocol version 6, dated 11 November, 2016. To date, five participants have been enrolled in the study. The study has been approved by NRES Committee South Central—Oxford A REC (Ref: 13/SC/0267) and is registered at clinicaltrials.gov (NCT01924598).

## Ethics Statement

Ethics approval and consent: this study will be carried out in accordance with the recommendations of NRES: South Central—Oxford A Research Ethics Committee (REC) Ref: 13/SC/0267. Informed written consent will be taken care by RP, in accordance with the Declaration of Helsinki. Consent can be freely withdrawn at any point with no effect on their usual clinical care.

## Author Contributions

RP designed the study, wrote the protocol, information sheets, ethical applications and grant applications and led on all psychiatric aspects of the study, in liaison with TA who contributed all surgical detail and led on all surgical aspects of the study. RP and JS will acquire and analyse the data. RP and JS drafted the manuscript. All authors read and approved the final manuscript.

## Conflict of Interest Statement

The patients will experience excellent follow-up quality clinical care from the neurosurgical DBS team irrespective of their trial involvement. The CI on the study is not responsible for the ongoing clinical psychiatric care of the patient, and therefore conflicts of interest should not arise. Involvement in this trial will not affect the choice of operation or longer-term stimulation parameters. There are no commercial conflicts of interest as there is no financial gain for researchers or patients.

## Footnotes

^1^University of Oxford. Available: http://researchsupport.admin.ox.ac.uk/ctrg

## Abbreviations

ALIC, Anterior Limb Internal Capsule; AN, Anorexia Nervosa; BMI, Body Mass Index; CANTAB, Cambridge Neuropsychological Test Automated Battery; CI, Chief Investigator; CTRG, Clinical Trials & Research Governance, University of Oxford; DBS, Deep-brain stimulation; DTI, Diffusion Tensor Imaging; FMRIB, fMRI Brain Imaging Centre, Oxford (John Radcliffe Hospital); HRA, Health Research Authority; IRAS, The Integrated Research Application System; NAcc, Nucleus Accumbens; NRES, National Research Ethics Service; OHBA, Oxford Centre for Human Brain Activity; PI, Principal Investigator; PIS, Patient Information Sheet; R&D, NHS Trust R&D Department; REC, Research Ethics Committee; RSN, Resting-State Network; SAE, Serious Adverse Event; SE-AN, Severe and Enduring Anorexia Nervosa; VS, Ventral Striatum.
